# Supplementary material for: Optimization of Imputation Strategies for High-Resolution Gas Chromatography–Mass Spectrometry (HR GC–MS) Metabolomics Data
Source: Metabolites. 2022 May 11;12(5):429. doi: 10.3390/metabo12050429 (PMC9144635; doi:10.3390/metabo12050429)
Supplement: Supplementary file 1 [file metabolites-12-00429-s001.zip › metabolites-1692346-supplementary.pdf]

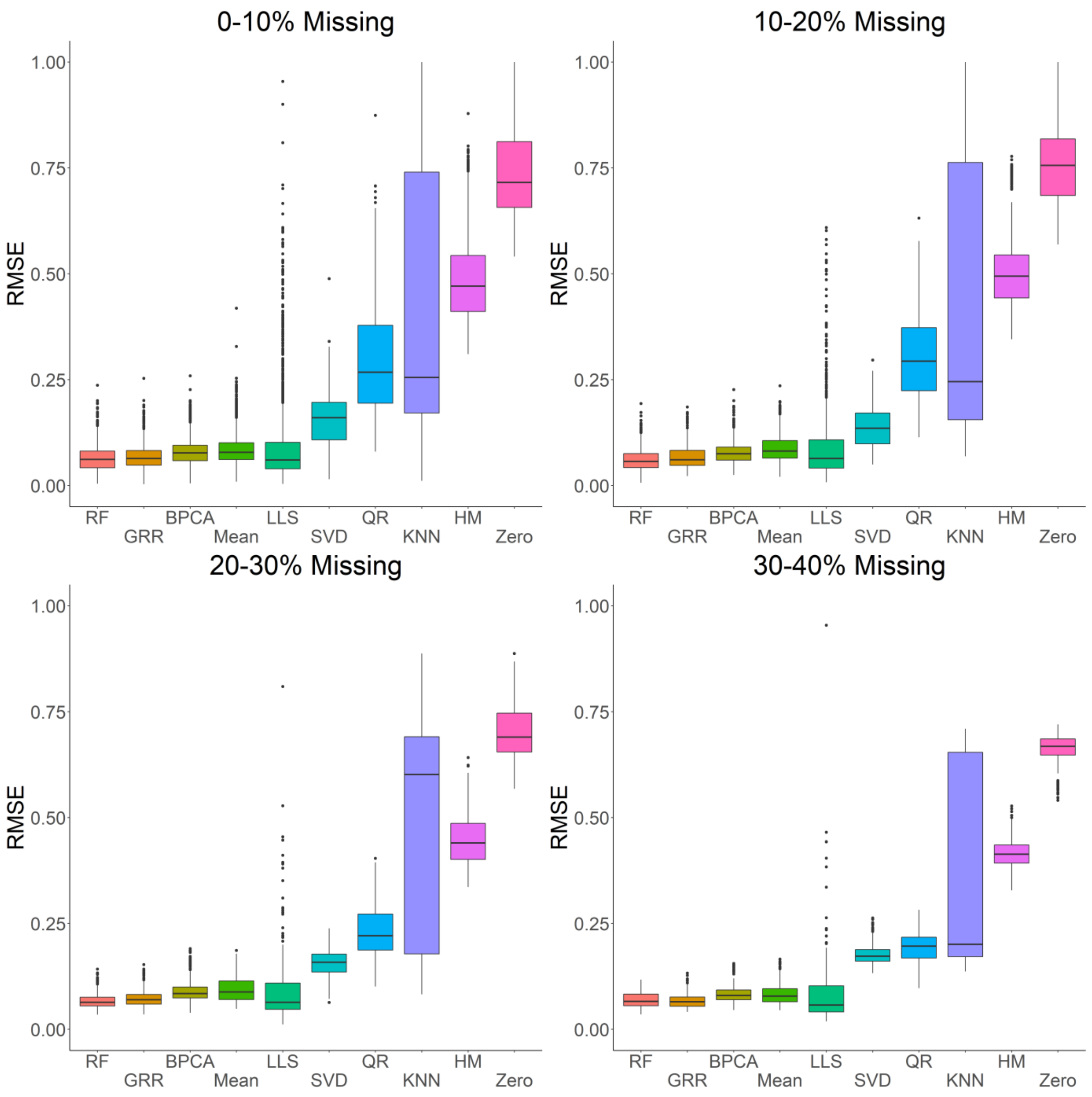

Figure S1. Initial evaluation of mixture of missingness types (MCAR-MAR-MNAR)

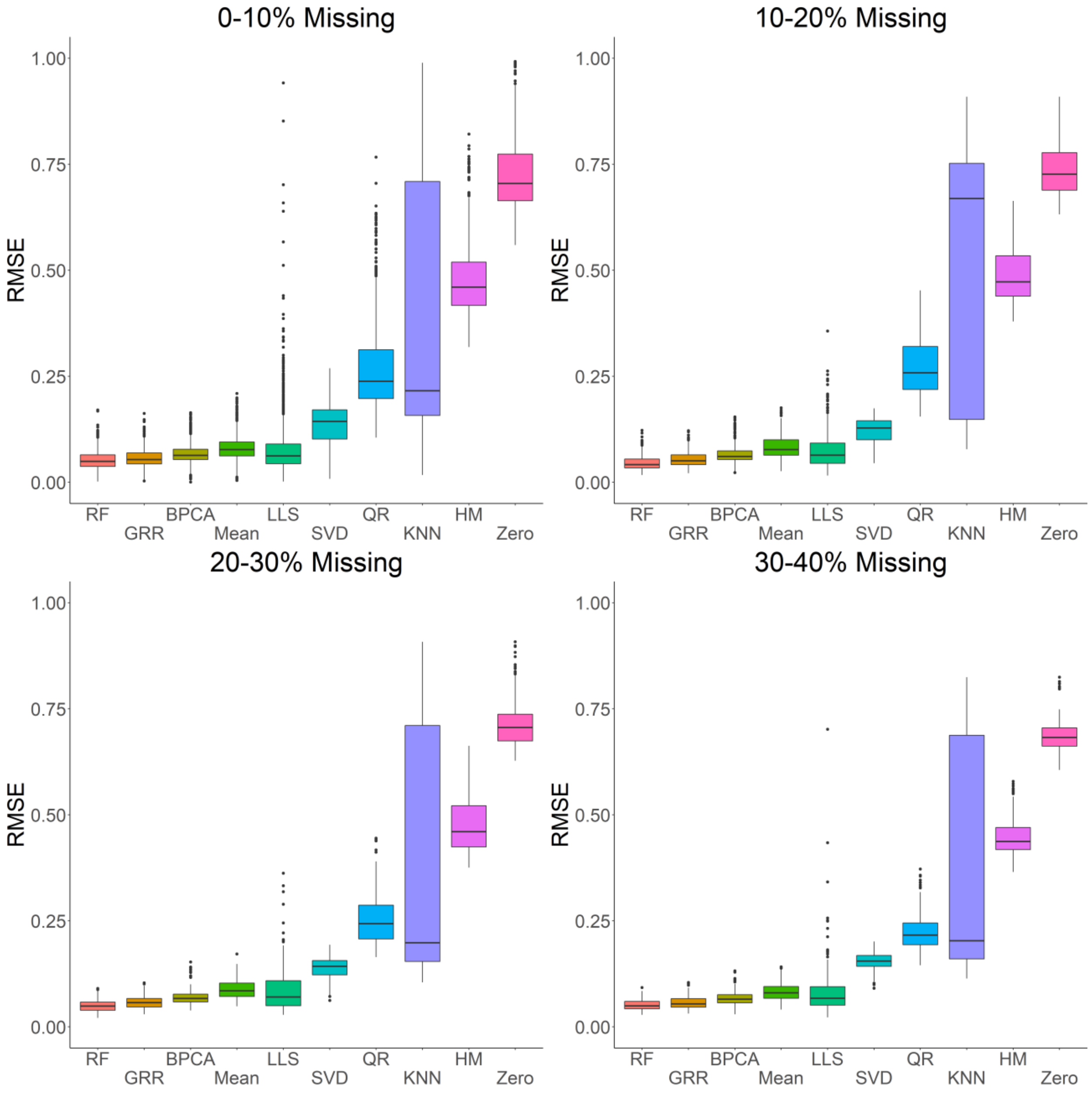

Figure S2. Initial evaluation of MAR missingness type

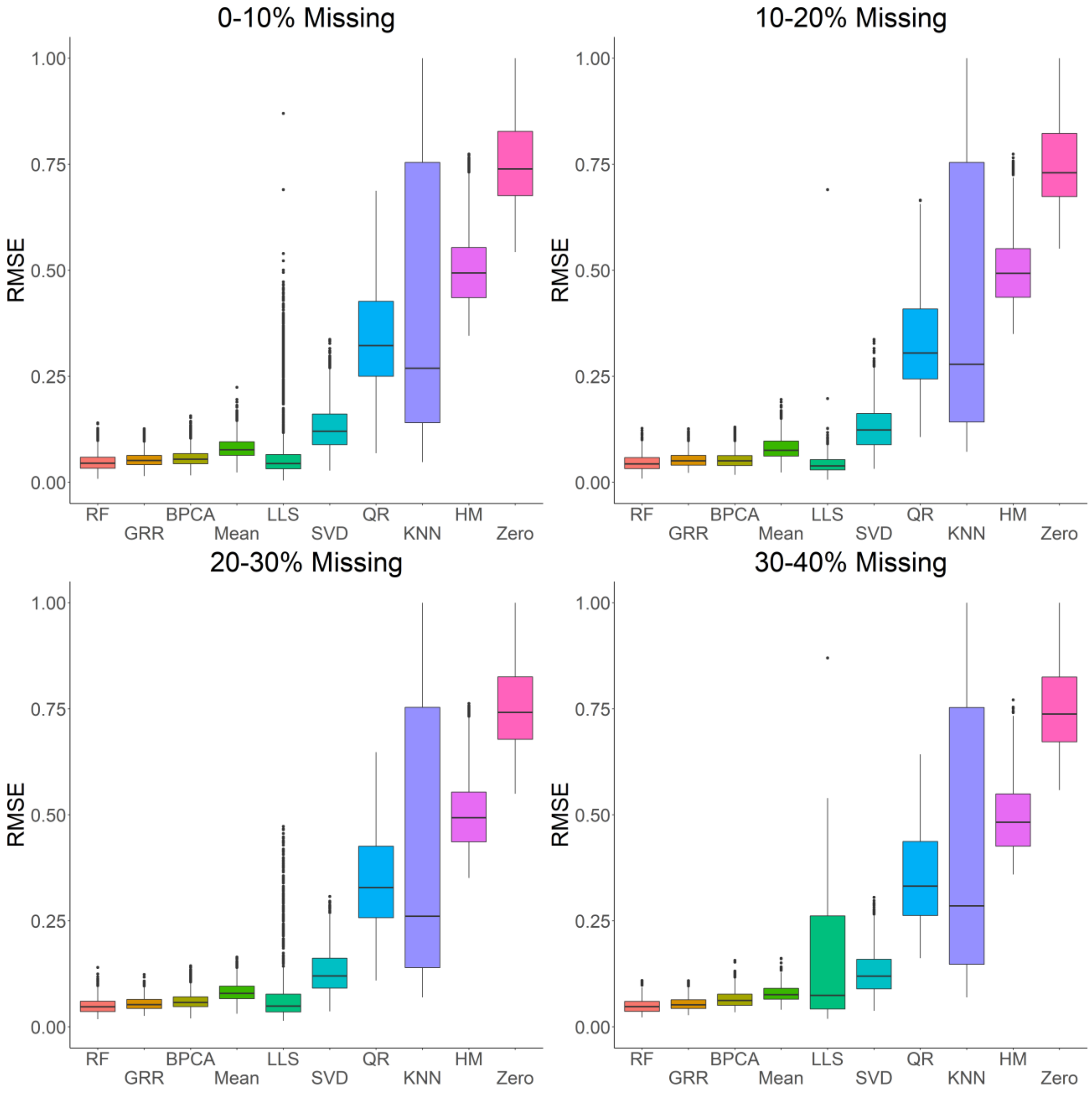

Figure S3. Initial evaluation of MCAR missingness type

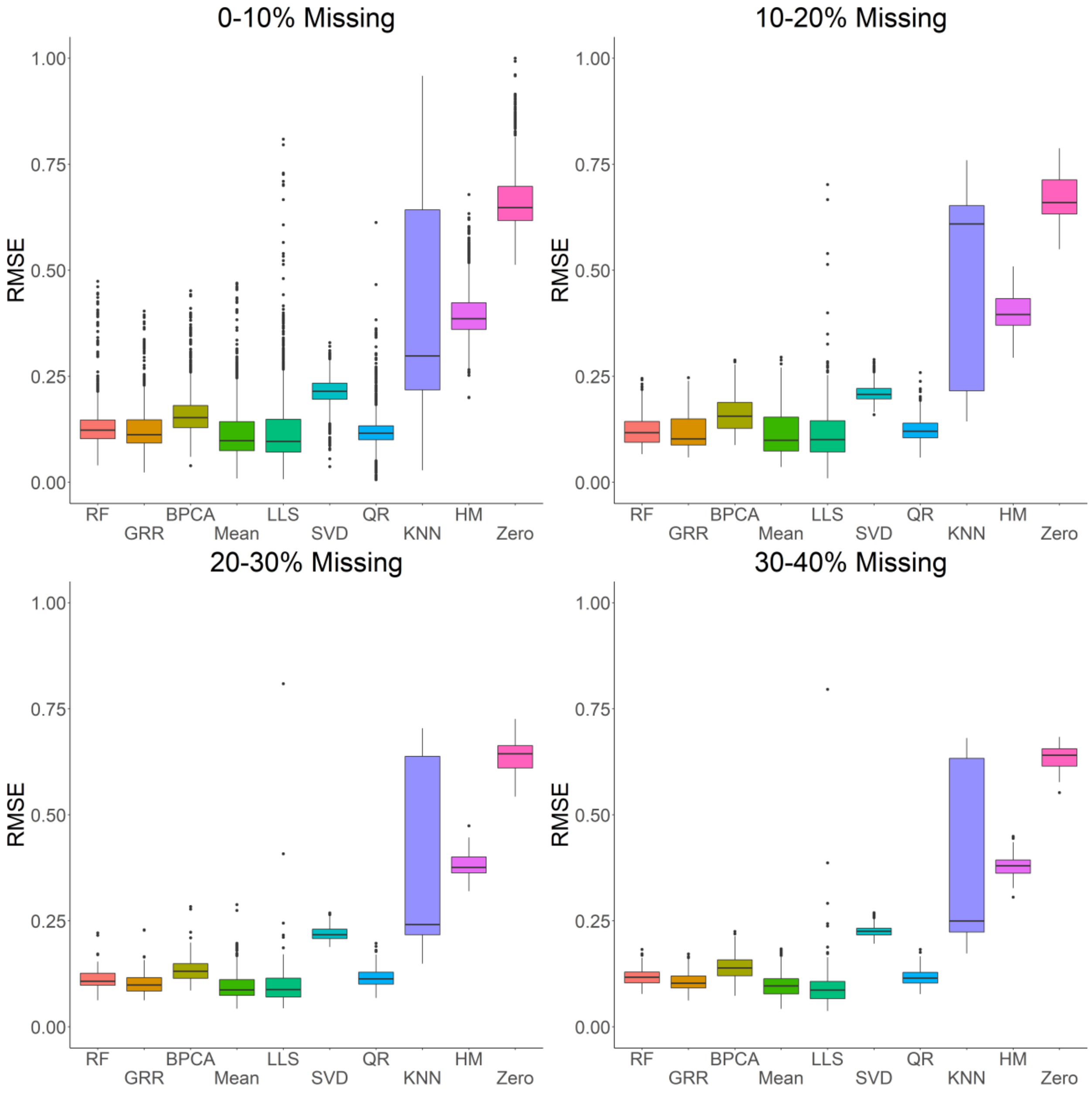

Figure S4. Initial evaluation of MNAR missingness type

Table S2. Each of the various imputation methods are listed with their respective R package names and parameters.

Imputation Method	R Package	Parameters
Random Forest (RF)	missForest (v1.4)	maxiter=10, ntree=100, default
Glmnet Ridge regression (GRR)	DreamAI	k=10, method="RegImpute", default
Bayesian Principle Component Analysis (BPCA)	pcaMethods (v 1.64)	allVariables=TRUE, default
Mean	base R (v 4.1.1)	-
Local least squares (LLS)	pcaMethods (v 1.64)	k=3, allVariables=TRUE, default
Singular value decomposition (SVD)	bcv (v 1.0.1.4)	Default
Quantile regression(QR)	imputeLCMD (v 2.0)	tune.sigma=1, default
k-nearest neighbor (KNN)	imputation (v 2.0.3)	k=3, default
Half Minimum (HM)	base R (v 4.1.1)	-
Zero	base R (v 4.1.1)	-
